# Functionalization of Single and Multi-Walled Carbon Nanotubes with Polypropylene Glycol Decorated Pyrrole for the Development of Doxorubicin Nano-Conveyors for Cancer Drug Delivery

**DOI:** 10.3390/nano10061073

**Published:** 2020-05-31

**Authors:** Chiara Pennetta, Giuseppe Floresta, Adriana Carol Eleonora Graziano, Venera Cardile, Lucia Rubino, Maurizio Galimberti, Antonio Rescifina, Vincenzina Barbera

**Affiliations:** 1 Department of Chemistry, Materials and Chemical Engineering “G. Natta”, Politecnico di Milano, Via Mancinelli 7, 20131 Milano, Italy; chiara.pennetta@polimi.it (C.P.); luciarita.rubino@polimi.it (L.R.); maurizio.galimberti@polimi.it (M.G.); 2 Department of Drug Sciences, University of Catania, Viale Andrea Doria 6, 95125 Catania, Italy; giuseppe.floresta@unict.it; 3 Department of Biomedical and Biotechnological Science, Section of Physiology, University of Catania, Via Santa Sofia 97, 95123 Catania, Italy; acegraz@unict.it (A.C.E.G.); cardile@unict.it (V.C.)

**Keywords:** carbon nanotubes, pyrrole, cancer, doxorubicin, drug delivery systems

## Abstract

A recently reported functionalization of single and multi-walled carbon nanotubes, based on a cycloaddition reaction between carbon nanotubes and a pyrrole derived compound, was exploited for the formation of a doxorubicin (DOX) stacked drug delivery system. The obtained supramolecular nano-conveyors were characterized by wide-angle X-ray diffraction (WAXD), thermogravimetric analysis (TGA), high-resolution transmission electron microscopy (HR-TEM), and Fourier transform infrared (FT-IR) spectroscopy. The supramolecular interactions were studied by molecular dynamics simulations and by monitoring the emission and the absorption spectra of DOX. Biological studies revealed that two of the synthesized nano-vectors are effectively able to get the drug into the studied cell lines and also to enhance the cell mortality of DOX at a much lower effective dose. This work reports the facile functionalization of carbon nanotubes exploiting the “pyrrole methodology” for the development of novel technological carbon-based drug delivery systems.

## 1. Introduction

*sp*^2^ carbon allotropes are fascinating materials. The discovery of fullerenes [1] “stimulated the creativity and imagination of scientists and paved the way to whole new chemistry and physics of nanocarbons” [2]. Within these materials, carbon black [3] is one of the ten most important chemical products, and recently novel families of *sp*^2^ carbon allotropes have become of great interest: carbon nanotubes), both single (SWCNT) [3,4] and multi-walled (MWCNT) [5,6], graphene [7,8,9,10] or graphitic nanofillers made by few layers of graphene [11,12,13,14].

CNT have a peculiar combination of electrical, thermal, and mechanical properties [15,16,17,18]. CNT find applications as superconductors [19], electrochemical capacitors [20], electromechanical actuators [21], photovoltaic devices [22,23], nanowires [24], in nanocomposite materials [25,26,27] and in medicinal chemistry [28]. CNT are emerging nanomaterials with great potential for diagnostic and therapeutic applications in medicine [28,29,30]. In this field, the biocompatibility of CNT is a hugely important aspect that has to be considered. In this direction, Prato and colleagues have reported some basic rules in the design of functionalized CNT to avoid deposition in specific tissues [28]. In particular, it was revealed that functionalized SWCNT are degraded inside cells such as neutrophils and macrophages in relation to the type of functional groups chemically introduced on the surface of CNT [31].

In the design of a potential CNT-based carrier, it is important to consider that: (i) they are usually available as highly entangled bundles; (ii) their dispersion in a matrix or a solvent is indeed very difficult. Moreover, the compatibility of CNT with the matrix depends on the solubility parameters of tubes and matrix. An effective way to overcome these problems is the functionalization of CNT, and several ways of achieving this have thus been developed. Functionalization of *sp*^2^ carbon allotropes can be classified as covalent [32,33] and non-covalent [34,35]. Some authors reported a sustainable functionalization method that is based on the principles of green chemistry [36], based on easily available chemicals, ideally biosourced [36,37] and economically feasible.

Moreover, the main goal was to identify a method able to promote the functionalization of most, if not all, the families of *sp*^2^ carbon allotropes. Graphene layers have been functionalized with pyrrole compounds obtained from the *Paal Knorr* reaction of a primary amine with a diketone, 2,5-hexanedione (HD). The selected *sp*^2^ carbon allotrope was functionalized by simply mixing it with the pyrrole compound (PyC) and giving either mechanical or thermal energy [37]. In the latter case, it was shown [37] that the graphene layers constituting the bulk structure of the carbon substrate remained substantially unaltered. Very high functionalization yields were reported [37]; in some cases, the functionalization achieved was from 80% to 95%. Studies on the reaction mechanism led to the hypothesis [38] that covalent bonds are formed between the carbon substrate and the pyrrole compound, with the occurring of a domino reaction: carbocatalyzed oxidation of the pyrrole compound and subsequent *Diels*-*Alder* cycloaddition. Preliminary indications have been reported [39] that the functionalization with different pyrrole compounds leads to the modification of the solubility parameter of graphene layers in a broad range of values.

This manuscript reports on the functionalization of single and multiwalled carbon nanotubes from now on indicated as CNT with a pyrrole compound obtained from HD and an amine-terminated poly(propylene glycol). Synthesis of the *O*-(2-(2,5-dimethyl-1*H*-pyrrol-1-yl) propyl)-*O*′-(2-methoxyethyl)polypropylene glycol (pyrrole polypropylene glycol, PPGP) is presented, the functionalization of CNT is described, and the main characteristics of the CNT/PPGP adducts are discussed. Two different adducts were prepared: the supramolecular adduct (CNT/PPGP_s_) and the covalent adduct (CNT/PPGP_c_). Characterization of the adducts was carried out employing thermogravimetric analysis (TGA), wide-angle X-ray diffraction (WAXD), high-resolution transmission electron microscopy (HR-TEM), and Fourier transform infrared spectroscopy (FT-IR). The ability of PyC to modify the solubility parameter of CNT was investigated. Based on the solubility parameter study and the easy functionalization procedure, the pyrrole ring was selected as the reactive moiety. The presence of the pyrrole at the end of the polymer chain allows obtaining a better conjugation with CNT respect to the polyether chain without a functionalizing molecule at the end.

The formation of the ternary nano complexes carbon nanotubes/polypropylenglycolpyrrole/doxorubicin (CNT/PPGP/DOX) is also reported. The supramolecular interactions between CNT/PPGP and DOX were studied by monitoring the emission and the absorption spectra of the drug employing fluorescence and Ultraviolet-visible (UV-Vis) spectrophotometries, respectively, and by molecular dynamics simulations. The ability of DOX to interact non-covalently with pristine and functionalized CNT and the evaluation of their capability to kill human melanoma and lung cancer cells is also reported. The release of DOX at slightly acidic pH was observed to be fast for MWCNT/PPGPc and slow for MWCNT/PPGPs and MWCNT. Preliminary biological studies revealed that two of the synthesized nano-vectors are effectively able to release the drug in situ.

## 2. Experimental Part

### 2.1. Materials

Reagents and solvents are commercially available and were used without any further purification: Methanol, 2-propanol, ethyl acetate, propylene glycol, dichloromethane, xylene, toluene, *n*-hexane, DOX hydrochloride, *O*-(2-aminopropyl)-*O*′-(2-methoxyethyl)polypropylene glycol (PPGA) (average M_n_ ≈ 600) and deuterated chloroform (CDCl_3_) were purchased from Sigma-Aldrich Merck KGaA group, (Darmstadt, Germany). Carbon nanotubes (CNT) were the *sp*^2^ carbon allotropes used in this work: multi-wall carbon nanotubes (MWCNT) were NANOCYL^®^ NC7000™ series from Nanocyl SA (Sambreville, Belgium), with a carbon purity of 90%, average length of about 1.5 µm and BET surface area (Brunauer Emmett Teller surface area) of 275 m^2^/g; single-wall carbon nanotubes (SWCNT) were TUBALL™ from OCSiAl (Leudelange, Grand-Duché de Luxembourg) with a carbon purity > 85%, average length of about 5 µm and BET surface area of 332 m^2^/g.

### 2.2. Synthesis of O-(2-(2,5-Dimethyl-1H-Pyrrol-1-Yl) Propyl)-O′-(2-Methoxyethyl)Polypropylene Glycol (Pyrrole Polypropylene Glycol, PPGP)

A quantits of 13.27 g of *O*-(2-Aminopropyl)-*O*′-(2-methoxyethyl)polypropylene glycol (PPGA, 0.02212 mol) and 2 g of 2,5-hexanedione (HD, 0.02212 mol) were poured in a 100 mL round-bottomed flask equipped with a magnetic stirrer. The mixture was then stirred (300 rpm) at 100 °C for 4 h. At the end of the reaction, the mixture still contained 2,5-hexanedione. The reagent was removed at reduced pressure (2 mbar, 25 °C) by using a Claisen apparatus (Colaver s.r.l, Vimodrone, Italy). The pure product was obtained with a yield of 64%. ^1^H NMR (CDCl_3_, 400 MHz); *δ* (ppm) = 1.13–1.15 (m, 74H); 1.47 (d, 3H); 2.25 (s, 6H); 3.32 (m, 4H); 3.32 (m, 4H); 3.42–3.78 (m, 34H); 3.66–3.5 (m, 55H); 3.74 (m, 3H); 4.35 (quintet, 1H); 5.68 (s, 2H); ^13^C NMR (CDCl_3_, 100 MHz); *δ* (ppm) = 127.0, 105.0, 80, 71.2, 70.0, 68.3, 62.0, 59.3, 31.0, 20.0, 13.2.

### 2.3. Preparation of CNT/PPGP Adducts

#### 2.3.1. Preparation of CNT/PPGP Supramolecular Adduct (CNT/PPGP_s_)

In a 250 mL round-bottomed flask CNT (500 mg) and acetone (20 mL) were put in sequence. The system was sonicated for 30 min, and then the pyrrole derivative (150 mg) was added into the flask. The suspension was sonicated again for 30 min. After solvent removal under reduced pressure, the mixture was quantitatively transferred in a funnel with a sintered glass disc, washed with acetone (100 mL), and then recovered and weighed.

The degree of functionalization was estimated employing TGA, determining the amount of pyrrole compound in the adduct after washing (mass losses for CNT and CNT/PPGP adducts are in Table 1), see Table 2: SWCNT/PPGP_s_ 5.48 w%; MWCNT/PPGP_s_ 3.00 w%.

#### 2.3.2. Preparation of CNT/PPGP Covalent Adduct (CNT/PPGP_c_)

In a 250 mL round-bottomed flask equipped with magnetic stirrer, we added CNT (500 mg) and 20 mL of acetone in sequence. The system was sonicated for 30 min, and then the pyrrole derivative (150 mg) was added into the flask. The suspension was sonicated again for 30 min. After solvent removal under reduced pressure, the CNT/PPGP mixture was poured in a round bottom flask and heated at 150 °C for 2 h. After this time, the mixture was quantitatively transferred in a funnel with a sintered glass disc, washed with acetone (100 mL), and then recovered and weighed.

The degree of functionalization was estimated employing TGA, determining the amount of pyrrole compound in the adduct after washing (mass losses for CNT and CNT/PPGP adducts are in Table 1), see Table 2: SWCNT/PPGP_c_ 5 w%; MWCNT/PPGP_c_ 7.00 w%.

### 2.4. Preparation of Carbon Nanotube/Pyrrole Polypropylene Glycol/Doxorubicin CNT/PPGP/DOX Ternary Nano Complexes

#### 2.4.1. General Procedure

DOX hydrochloride (9 mg) was stirred with the selected CNT/PPGP adduct (3 mg) dispersed in a pH 7.4 Phosphate Buffered Saline solution (PBS) (6 mL) and stirred for 16 h at room temperature. The product was collected by ultracentrifugation with PBS until the supernatant became colorless. The amount of unbound DOX was determined by measuring the absorbance at 490 nm of the supernatant after centrifugation see Table S1. The CNT/PPGP/DOX nano complex dispersions in PBS were also analyzed by fluorescence spectrophotometry: dispersions were placed using a Pasteur pipette (Colaver s.r.l, Vimodrone, Italy) in a triangular quartz cuvette. A Jasco FP-6600 Spectrofluorometer (JASCO corporation, Tokyo, Japan) was used to perform the fluorescence measurements. The fluorescence detector was set at an excitation wavelength of 480 nm, and fluorescence spectra in the range of 500–700 nm were collected.

#### 2.4.2. DOX Calibration Curve by UV-Vis Spectroscopy

A stock PBS solution of DOX hydrochloride (1 mg/mL) at pH 7.4 was prepared. The obtained solution was then diluted, and UV-Vis measurements were performed. The absorbance of these solutions was measured at 490 nm of maximum absorbance, using a 1 cm quartz cuvette.

#### 2.4.3. DOX Release From CNT Nano Complexes

CNT/DOX and CNT/PPGP/DOX in acetate buffer (pH 5.5) were loaded in Spectra/Por^®^ Dialysis Membrane (10K MWCO, nominal flat width 24 mm, diameter 15 mm wet in 0.1% sodium azide) (Thermo Fisher Scientific Inc., Waltham, MA, USA). Each dialysis bag was then allowed to stand for 72 h in acetate buffer solution. The release of DOX was checked after 24, 48, and 72 h through UV-Vis spectroscopy. The same experiment conducted in PBS showed that DOX remained bound to CNT due to the stability of the drug at pH 7.4.

### 2.5. Characterization of Pristine CNT, CNT/PPGP Adducts and CNT/PPGP/DOX Ternary Nano Complexes

#### 2.5.1. Fourier Transform Infrared Spectroscopy (FT-IR)

The IR spectra were recorded in transmission mode (128 scans and 4 cm^−1^ resolution) using a Thermo Electron Continuum IR microscope coupled with an FTIR Nicolet Nexus spectrometer. A small portion of the dry solid material was placed in a diamond anvil cell (DAC) and analyzed in transmission mode.

#### 2.5.2. Thermogravimetric Analysis (TGA)

TGA test under N_2_ flowing (60 mL/min) was performed with a Mettler TGA SDTA/851 instrument according to the ISO9924-1 standard method. Samples (10 mg) were heated from 30 to 300 °C at 10 °C/min, kept at 300 °C for 10 min, and then heated up to 550 °C at 20 °C/min. After being maintained at 550 °C for 15 min, they were further heated up to 700 °C and kept at 700 °C for 30 min under air flowing (60 mL/min).

#### 2.5.3. High-Resolution Transmission Electron Microscopy (HR-TEM)

HR-TEM investigations on CNT and CNT/PPGP adducts were carried out with a Philips CM 200 field emission gun microscope operating at 200 kV. Few drops of the water suspensions were deposited on 200 mesh lacey carbon-coated copper grid and air-dried for several hours before analysis. During the acquisition of HR-TEM images, performed with low beam current densities and short acquisition times, the samples did not undergo structural transformation. The Gatan Digital Micrograph software (GMS 3, Gatan, Inc., Pleasanton, CA, USA) was used to estimate in HRTEM micrographs the number of stacked graphene layers and the dimensions of the stacks.

#### 2.5.4. Wide-Angle X-Ray Diffraction

Wide-angle X-ray diffraction patterns were obtained in reflection, with an automatic Bruker D8 Advance diffractometer (Bruker Corporation, Billerica, MA, USA), with nickel filtered Cu–K*_α_* radiation. Patterns were recorded in 10–80° as the 2*θ* range, being 2*θ* the peak diffraction angle. Details can be found in Section S1.

### 2.6. Preparation and Characterization of Dispersions of CNT/PPGP Adducts in Different Solvents-Evaluation of Solubility Parameters

#### 2.6.1. Preparation of Water Dispersions of CNT/PPGP Adducts

General procedure: water dispersions of CNT/PPGP adducts were prepared at different concentrations: 1 mg/mL; 0.5 mg/mL; 0.1 mg/mL; 0.05 mg/mL; 0.01 mg/mL; 0.005 mg/mL; 0.001 mg/mL. Each dispersion was sonicated for 1 min using an ultrasonic bath (260 W). The dispersion (10 mL) of each sample was put in a Falcon™ 15 mL Conical Centrifuge Tubes and centrifuged at 6000 rpm for 30 min. UV-Vis measurement was performed immediately after sonication or centrifugation, and also after 3 days. A Hewlett Packard 8452A Diode Array Spectrophotometer (Hewlett-Packard, Palo Alto, CA, USA) was used to perform the absorption measurements. The dispersions were placed using a Pasteur pipette in cuvettes with an optical path of 1 cm (about 3 mL per cuvette). The obtained UV-visible spectrum reports absorption as a function of radiation wavelength in the range of 200–750 nm.

#### 2.6.2. Calculation of the Hansen Solubility Sphere and Hansen Solubility Parameters

The calculation of the *Hansen* solubility parameters (HSP) for CNT was performed by applying the Hansen Solubility Sphere representation of miscibility. The idea at the basis of this geometrical approach is the calculation of the cohesive energy density (U_T_/V) of a compound as the sum of three interaction contributions: non-polar Van der Waals forces (*δ_D_*), polar (*δ_P_*) and hydrogen bonding (*δ_H_*). Details can be found in Section S2.

### 2.7. Biological Studies

#### 2.7.1. Cell Cultures

The M14 human melanoma and the A549 human lung adenocarcinoma cell lines have been used in the present investigation. Both cell lines were routinely maintained as previously described [40,41] in a humidified atmosphere of 5% CO_2_ in a water-jacketed incubator at 37 °C. The base media were Roswell Park Memorial Institute (RPMI) 1640 for M14 cells, and Dulbecco’s modified Eagle’s medium (DMEM) for A549 cells. The complete growth media were obtained by supplementation with 10% (by volume) heat-inactivated fetal bovine serum, 2 mM glutamine, 100 units/mL penicillin, and 100 μg/mL streptomycin (Thermo Fisher Scientific, Milan, Italy). The cells were subcultured before reaching confluence, using a 0.25% trypsin-EDTA solution (Carlo Erba, Milan, Italy).

#### 2.7.2. Cell Viability Assay

Cells were seeded in 96-well cell culture plates at a density of 2 × 10^4^ cells/well (200 µL/well) for in vitro cell viability assay. After overnight incubation to allow the attachment of cells, the resulting monolayers were incubated with free DOX hydrochloride or into both unloaded and loaded CNT for 48 h.

In these experiments, all CNT samples were bath sonicated for 5 min in culture medium in order to obtain a homogeneous dispersion at 1 mg/mL. The stock dispersions were diluted in culture medium to get the desired concentrations referred and normalized to the amount of loaded DOX hydrochloride into each sample. After the time of incubation, the cells were carefully washed to remove the non-internalized nanotubes, and cytotoxicity was determined by 3-(4,5-dimethyl-2-thiazolyl)-2,5-diphenyl-2*H*-tetrazolium bromide (MTT) assay according to a previously published protocol [42]. The formazan was solubilized in DMSO (Sigma-Aldrich, Italy) and spectrophotometrically quantified at *λ* = 550 nm by a microplate reader (Titertek Multiscan, DAS, Milan, Italy).

#### 2.7.3. Statistical Analysis

The results were expressed as mean ± s.e.m. based on data derived from three independent experiments run in triplicate. Statistical analysis of results was performed using Student’s *t*-test and one-way ANOVA test plus Dunnett’s test by the statistical software package SYSTAT, version 11 (Systat Inc., Evanston, IL, USA).

### 2.8. Computational Details

#### 2.8.1. Models Preparation

SWCNT and MWCNT were generated from the Nanotube Modeler package [43] (v. 1.8, JCrystalSoft) with the armchair arrangement, all open and hydrogen-terminated, trying to be faithful, as diameter and number of walls, to the values reported in the literature [34]. The SWCNT structure with a length of 50 Å and the chiral vectors m = 13, and n = 13 (13,13) with a diameter of 17.640 Å, and the MWCNT structure with a length of 10 Å and the chiral vectors (25,25) for an inner diameter of 33.924 Å and (70,70) for an outer diameter of 94.987 Å were used as the model for the CNT/PPGP/DOX drug carrier systems. The initial structure of DOX was obtained from the DRUGBANK server [44], whereas that of PPGP was manually built.

#### 2.8.2. Molecular Dynamics Simulations

The molecular dynamics simulations of the CNT/PPGP/DOX supramolecular systems (SSy) were performed with the YASARA Structure package (v. 19.10.23). A periodic simulation cell with boundaries extending 10 Å [45] from the surface of the CNT or CNT/PPGP_c_ was employed. For the two SWCNT systems (1066 carbon atoms), we used 1 PPGP and 45 DOX molecules (SSy1,2, see paragraph 3.4), whereas for the two MWCNT systems (8550 carbon atoms), we used 12 PPGP and 369 DOX molecules for PPGP_s_ arrangement, and 5 PPGP and 354 DOX molecules for the PPGP_c_ one (SSy3,4, see paragraph 3.4). For the SWCNT/PPGP_c_ system, the one PPGA molecule was covalently linked about at the center of the CNT structure, whereas for the MWCNT/PPGP_c_ system, the five PPGA molecules were covalently linked, approximately regularly spaced, around the outer circumference of the CNT structure. Details can be found in Section S3.

## 3. Results and Discussion

### 3.1. Modification of the CNT with a Pyrrole Decorated Polypropylene Glycol

**Figure 1:**
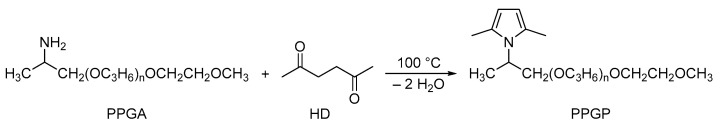
Synthesis of *O*-(2-(2,5-dimethyl-1*H*-pyrrol-1-yl) propyl)-*O*′-(2-methoxyethyl)polypropylene glycol (Pyrrole polypropylene glycol, PPGP).

Functionalization of carbon nanotubes, either single (SWCNT) and multi-walled (MWCNT), was realized by using a pyrrole terminated polymer (Pyrrole polypropylene glycol, PPGP) as a modifying agent. PPGP was synthesized through the *Paal*-*Knorr* reaction [34,39], by reacting the amino terminated polymer *O*-(2-Aminopropyl)-*O*′-(2-methoxyethyl)polypropylene glycol (PPGA) with HD as reported in the experimental part and summarized in Figure 1.

The reaction was performed in the absence of catalysts by adopting experimental conditions inspired by the basic principles of green chemistry [36]. In particular, acidic catalysts and toxic solvents traditionally used for the reactions of primary amines with carbonyl compounds were avoided. In previous works [34,37], it was shown that the reaction of 2-amino-1,3-propanediol (serinol) with 2,5-hexanedione led to a 1,3-bis-oxazolidine compound, which was then converted into a pyrrole compound only by heating. The reaction of PPGA with HD was performed by heating at 100 °C. Water was the only co-product of the reaction, which was characterized by a high atom economy of 88.2% and by a yield of 64%. The chemical structure of PPGP was confirmed through ^1^H NMR spectroscopy Figure S1. PPGP appears as a light-yellow liquid with slightly higher viscosity at room temperature and normal pressure. PPGP contains a long polyether chain that could favor the compatibility with polar surroundings such as water, alcohols, and polar polymer matrices. PPGP also contains the pyrrole ring, which could favor *π*–*π* interaction with the aromatic rings of the *sp*^2^ carbon allotropes.

**Figure 2:**
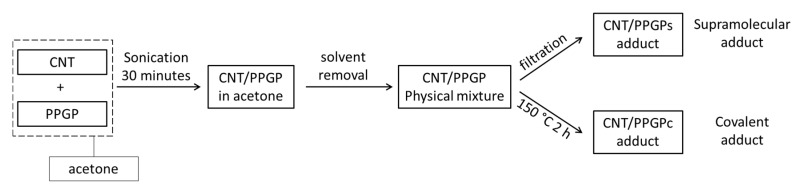
Block diagram summarizing the preparation of CNT/PPGP adducts.

As stressed in the introduction, pyrrole compounds (PyC) were shown to be able to form stable adducts with graphitic substrates [37,38]. In this work, reactions between CNT and PPGP were performed by adopting the experimental frame summarized in Figure 2.

In the experimental part, the functionalization of CNT by using PPGP as the modifying agent is described extensively. In brief, CNT were initially sonicated in the presence of the pyrrole terminated polymer (PPGP) in acetone, leading to the formation of the CNT/PPGP_s_ adducts (supramolecular adduct) after removal of the solvent and washing with acetone. However, in a one-pot process involving the direct thermal treatment of the physical mixture (150 °C, 2 h), the covalent adduct CNT/PPGP_c_ was easily obtained. No further optimization of the reaction conditions was performed.

**Figure 3:**
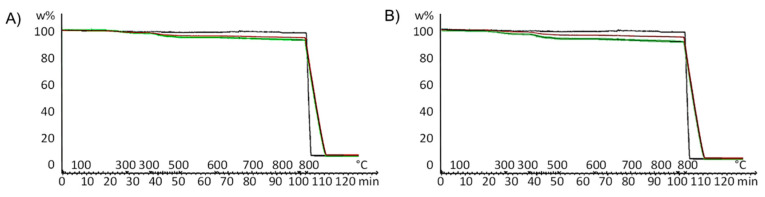
Thermogravimetric analysis (TGA) curves of: (**A**) single-wall carbon nanotubes (SWCNT) (black line), SWCNT/PPGP_s_ (green line), and SWCNT_c_ (red line); (**B**) multi-walled carbon nanotubes(MWCNT) (black line), MWCNT/PPGP_s_ (green line), and MWCNT_c_ (red line).

In order to establish the efficiency of the functionalization, the CNT/PPGP powder taken from the flask was extracted in *Soxhlet* with acetone until PPGP was undetectable in the washing solvent. TGA was performed on CNT and the CNT/PPGP adducts before and after washing with acetone. As described in detail in the experimental part, the TGA was carried out under nitrogen up to 700 °C and under oxygen up to 800 °C. Thermographs of both single and multiwall CNT and CNT/PPGP adducts are shown in Figure 3. The values of mass losses are reported in Table 1.

The mass loss at T < 150 °C was attributed to absorbed low molar mass molecules, mainly water. The decomposition profile for all the samples, pristine CNT and CNT/PPGP adducts, reveals two main steps in the temperature range from 150 °C to 700 °C, which can be attributed to the decomposition of alkenyl-, oxygen- and nitrogen-containing functional groups. New final decomposition occurs at T > 700 °C, due to reaction with the oxygen of the graphitic structure. The amount of PPGP in the adduct was estimated by evaluating the mass loss in the temperature range from 150 °C to 700 °C.

By comparing data referring to CNT and CNT/PPGP adducts, it appears that the mass loss is more significant for the latter ones. It can also be observed that CNT/PPGP adducts containing the polymeric chain give rise to a more substantial mass loss below 150 °C, and this could be due to a more considerable amount of absorbed water.

The degree of functionalization and the functionalization yield were calculated through Equations (1) and (2), and their values are reported in Table 2.

(1) Degree of functionalization (%)=100·[(wt.% loss CNT/PPGP)−(wt.% loss CNT)]

(2) $$Functionalization\;yield\;\left(\%\right)=100\cdot \frac{PPGP\;mass\;\%\;in\;(CNT/PPGP\;adduct)\;after\;acetone\;washing}{PPGP\;mass\;\%\;in\;(CNT/PPGP\;adduct)\;before\;acetone\;washing}$$

Although optimization of the reaction conditions was not performed, functionalization yield was high for all the CNT adducts except for the MWCNT/PPGP_s_ adduct, which was 56%. This significant difference in the functionalization efficiency between the covalent and the supramolecular MWCNT adducts occurs since the commercial MWCNT are usually much entangled. The covalent functionalization helps the disentanglement of MWCNT increasing the available surface, which allows a better interaction between the functionalizing molecule and the MWCNT. In the MWCNT supramolecular adducts, there is no such effect, and the carbon nanotubes remain much entangled.

Mass loss values in the range 150–900 °C were exploited to determine the degree of functionalization of each adduct, defined as the percent value of the difference between the mass loss of the CNT/PPGP and that of the pristine CNT in the studied temperature range.

Thermogravimetric experiments showed that the functionalization of the considered adduct with PPGP was successful and that the introduction of an appropriate amount of modifier was achieved. The characterization of the CNT/PPGP adducts was performed employing IR and WAXD spectroscopies.

**Figure 4:**
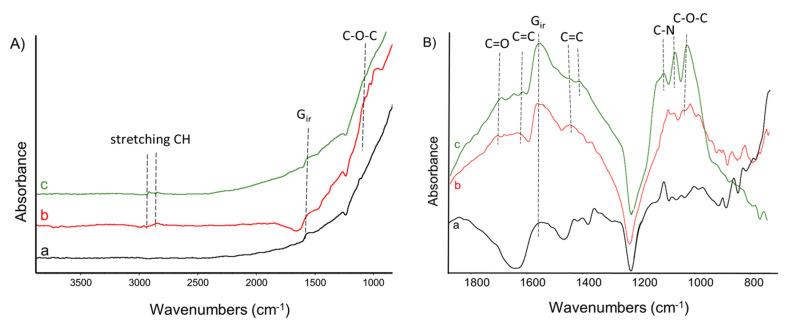
FT-IR spectra of MWCNT (**a**), MWCNT/PPGP_s_ (**b**), and MWCNT/PPGP_c_ (**c**). (**A**): 700–3900 cm^−1^ region; (**B**) fingerprint region 700–1800 cm^−1^ after baseline correction. Spectra are displayed with normalized intensity. Peaks discussed in the text are labeled.

Infrared spectroscopy allowed a qualitative check of the chemical nature of the attached molecule and was performed on pristine CNT and CNT/PPGP adducts after acetone extraction. In Figure 4A,B the IR spectra of CNT and CNT/PPGP adducts are reported. As explained in detail in the experimental part, IR spectra have been recorded in transmission mode using a diamond anvil cell (DAC) in order to avoid the absorption peaks of water molecules. The spectra were obtained from the absorption of very thin films of CNT powder, which are not transparent to the IR beam. Indeed, the *G*_IR_ absorption observed in the spectra at 1590 cm^−1^ is mostly due to the reflection from the graphitic planes. The intense light diffusion from the high surface area graphite (HSAG) particles is responsible for the increase of the absorbance in the spectra toward higher wavenumbers. Absorption spectra recorded on DAC are presented in the region 700–3900 cm^−1^ Figure 4A and in the fingerprint region 700–1800 cm^−1^ Figure 4B after baseline correction to make easier the comparison of the weak spectroscopic features. The low and broad vibrational signals floating on a rather steep background absorption and the chemical complexity of the samples severely limits a structural diagnosis through a detailed assignment of the spectral features. Thus, the vibrational analysis was based on the recognition of the functional groups based on correlative spectroscopic criteria [38]. The FT-IR spectra of MWCNT, MWCNT/PPGP_s_, and MWCNT/PPGP_c_ are reported in Figure 4. In particular, Figure 4A shows the spectra recorded in the region 700–3900 cm^−1^, while in Figure 4B the spectra are displayed in the fingerprint region, after baseline correction, to allow easier comparison. In Figure 4, the IR spectra of MWCNT (a) MWCNT/PPGP_s_ (b) and MWCNT/PPGP_c_ (c) are characterized by the presence of the typical strong feature at 1590 cm^−1^. The spectra of MWCNT/PPGP_s_ and MWCNT/PPGP_c_ are both dominated by the very strong absorptions characteristic of their functional groups. Both spectra are dominated by (i) the absorption near 1100 cm^−1^ assigned to the stretching of C–O–C groups and by (ii) the broad bands at 1650 cm^−1^ and 1400 cm^−1^ assigned to the C=C stretching of the pyrrole ring. All these vibrations are compatible with the presence of PPGP functionalities. In the spectrum of MWCNT/PPGP_s_, bands related to unreacted pyrrole compounds are present: C–N stretching (aromatic amines) from 1335–1250 cm^−1^.

As reported in the introduction, another aim of this study was to investigate the ability of PPGP to modify the solubility parameter of carbon nanotubes in order to preliminarily understand if the projected carrier could be able to interact with different surrounding. Dispersions of CNT/PPGP adducts cited in Table 1 were prepared in solvents having different solubility parameters: water, methanol, 2-propanol, acetone, ethyl acetate, propylene glycol, dichloromethane, xylene, toluene, and hexane. The stability of such dispersions was studied, as described in the experimental part. Visual inspection of the dispersions was carried out immediately after sonication. In Table S2, the result of these observations is qualitatively summarized with ‘good’ (meaning that a homogenous dispersion was observed) or ‘bad’ (the adduct either settled down or floated on the solvent) as indicators.

**Figure 5:**
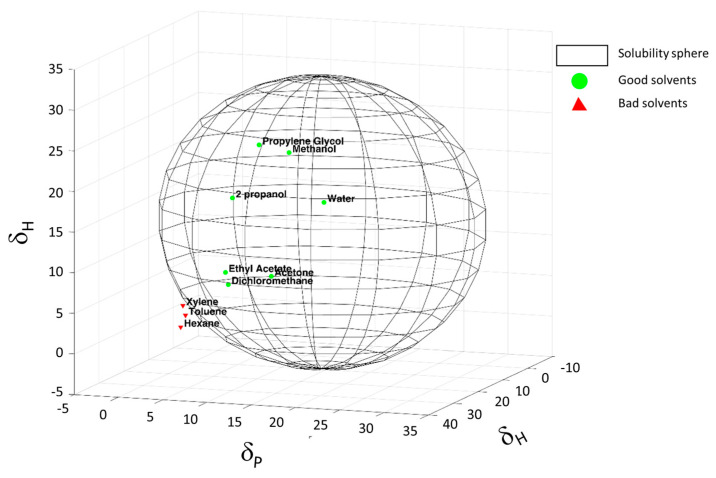
*Hansen* solubility sphere calculated for MWCNT/PPGP_c_ adduct. The green circles correspond to the suitable solvents (within the radius of interaction), the red triangles to the wrong solvents (outside the sphere).

The introduction of PPGP on the surface of the carbon allotropes allows the dispersion in polar environments thanks to the long polyether chain. The solubility sphere shown in Figure 5 was generated, as explained in the experimental part, to encompass the suitable solvents points and to exclude the wrong solvents, being centered on the solubility parameters of the MWCNT/PPGP_c_ adduct. Solubility parameters, *δ_D_*, *δ_P_*, and *δ_H_* values, of MWCNT/PPGP_c_ adduct, were estimated to be 11.48 MPa^0.5^, 15.40 MPa^0.5^, and 18 MPa^0.5^, respectively.

As it should be expected, considering the presence of such functional groups and a polyether long chain, water dispersions of CNT/PPGP adducts were easily prepared. Preparation and UV-Vis absorption data of water dispersions of CNT/PPGP adducts were reported and discussed in the following part.

**Figure 6:**
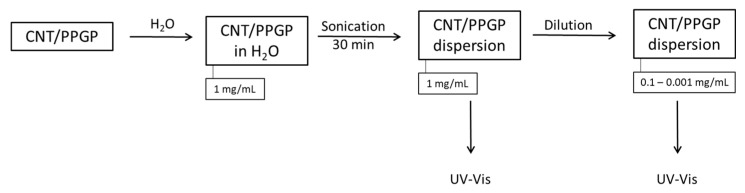
Block diagram summarizing the preparation procedure and characterization protocol adopted for UV-Vis analysis on CNT/PPGP adducts dispersions at several concentrations.

CNT/PPGP water dispersions with the following concentrations were prepared: 1, 0.5, 0.1, 0.05, 0.01, 0.005 and 0.001 mg/mL. The dispersions were then analyzed by observing their absorptions in the UV-Vis range. Details are reported in the experimental section for each CNT/PPGP adduct. The adopted procedure is summarized in the block diagram of Figure 6.

**Figure 7:**
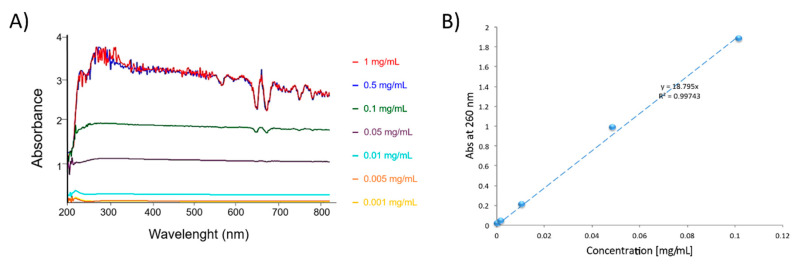
(**A**) Dependence of UV-Vis absorbance on the concentration of MWCNT/PPGP_c_ adduct in water after sonication; (**B**) Linear relationship between absorbance at 260 nm and concentration for MWCNT/PPGP_c_ adduct.

Adduct dispersions at different concentrations were prepared to investigate if the Lambert-Beer law is respected. If so, the CNT/PPGP adduct can be assumed to form a “solution-like” substance with water, thanks to the added polyether chain on its surface. Figure 7 shows the dependence of UV-Vis absorbance on the concentration of MWCNT/PPGP_c_ adduct in water after sonication (A) and the linear relationship between absorbance at 260 nm and concentration for MWCNT/PPGP_c_ (B).

Furthermore, curves related to the 0.005 and 0.001 mg/mL dispersions reveal the presence of a non-negligible absorbance; this suggests high stability of the functionalized fractionated adduct still in suspension, not observed in the case of non-functionalized CNT.

Absorption values corresponding to a wavelength of 260 nm for MWCNT-PPGP_c_ adduct curves were plotted as a function of concentration values. In Figure 7B, a linear correlation is reported.

Structure and morphology of the CNT and the ensuing CNT/PPGP adducts were investigated through WAXD and HR-TEM analysis. Figure S4 shows WAXD patterns for powders of MWCNT, MWCNT/PPGP_s_, and MWCNT/PPGP_c_. In pristine MWCNT Figure S4a, crystalline order in the direction orthogonal to structural layers is revealed by 002 reflection at 26.6°, which corresponds to an interlayer distance of 0.35 nm. By applying the *Scherrer* equation (Equation (S2)) to (002) reflection, the out of the plane (*D*_┴_) correlation length was calculated. From the values of *D*_┴_ and of the interlayer distance, the number of stacked layers was estimated to be about 12 for CNT. Reflections in the patterns of the *sp^2^* adducts samples Figure S4b,c remains at the same 2*θ* value. MWCNT and MWCNT/PPGP adducts present distances between the structural layers slightly more significant than those of ordered graphite samples (*d*_002_ = 0.335 nm). In all samples, (112) reflection is negligible.

**Figure 8:**
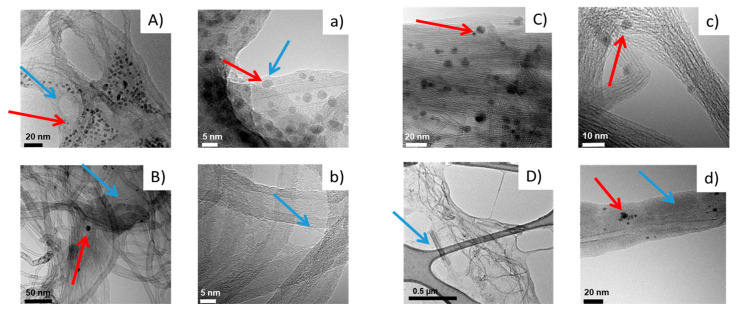
Micrographs of MWCNT/PPGP_s_ (**A**,**a**), MWCNT/PPGP_c_ (**B**,**b**), SWCNT/PPGP_s_ (**C**,**c**) and SWCNT/PPGP_c_ (**D**,**d**) adducts isolated from 1 mg/mL water dispersions. Micrographs are: low magnification bright-field TEM (**A**–**D**), HR-TEM images (**a**–**d**); (the light blue arrows indicate layers of organic substances; the red arrows indicate spherical organic aggregates).

High-resolution transmission electron microscopy (HR-TEM) was exploited to study the morphology of CNT/PPGP adducts. Various magnifications were adopted. Figure 8 shows HR-TEM micrographs of MWCNT/PPGP_s_ (A,a), MWCNT/PPGP_c_ (B,b), SWCNT/PPGP_s_ (C,c) and SWCNT/PPGPc (D,d) adducts, at lower (A,B,C,D) and at higher magnifications (a,b,c,d).

Micrographs at a lower magnification of each CNT/PPGP adduct in Figure 8 reveal that the length of CNT/PPGP adducts is of the same order of magnitude in samples isolated before and after PPGP treatments. This indicates that the chemical interaction with PPGP and the heating step for the preparation of the covalent adducts do not cause appreciable breaking of the nanotubes.

In the case of MWCNT/PPGP_s_ and SWCNT/PPGP_s_ adducts in water dispersions, micrographs at lower magnification in Figure 8A,C reveal carbon aggregates made by pseudo-spherical particles with an average size of about 5–10 nm. Figure 8B,b shows the MWCNT/PPGP_c_ covalent adduct. It seems that a layer of organic substance (indicated by the light blue arrow) adheres to the carbon allotrope. It appears that the organic substance is probably made by unreacted PPGP, which covers the surface. Also, in the case of MWCNT/PPGP_c_, a low quantity of spherical organic aggregates (indicated by the red arrow) were detected (average dimension~5–20 nm). It appears that spherical agglomerates are probably made by unreacted PPGP also in this case. It is known from the literature that macromolecules like polyether terminated with cationic or anionic functional groups can generate micelle or more in general supramolecular assembly structures [46].

Micrographs at higher magnification Figure 8a–d allow visualizing walls of nanotubes. In the case of SWCNT, the covalent treatment with PPGP led to a large CNT disentanglement, as shown by the lower number of CNT micrometric bundles and by the presence of individual tubes in a defined space, as observed in many HR-TEM images and represented in Figure 8. The micrograph in Figure 8d shows that the SWCNT skeleton remained intact after the treatment with PPGP oligomer. The CNT surface was thus decorated with PPGP chains, which form condensed polymer layers adhered to the CNT external surface, with a thickness from about 3 to about 10 nm.

Furthermore, a comparison between TEM micrographs at low magnifications of pristine CNT and PPGP adducts are reported in Figure S5. Figure S5a,b show that a high entanglement of nanotubes characterizes pristine MWCNT (a) and SWCNT (b). The functionalization with PPGP improves the disentanglement of CNT, allowing better processability of the carbon allotropes dispersions Figure S5c–f.

### 3.2. Preparation and Characterization of the Ternary Nano Complex CNT/PPGP/DOX

HR-TEM microscopy Figure 8 allows checking that both supramolecular adducts show on their surface micelle-like structures and adhered polymer chains, and to the contrary, that the covalent adducts show only a regular adherent polymeric layer. In both cases, the possibility of a drug loading seems possible via adsorption on the CNT surface. Previously, it has been shown that the mixing of the SWCNT with DOX leads to the absorption of DOX onto the outer sides of SWCNTs via *π*–*π* stacking interactions. It was reported that suitably functionalized SWNT and MWCNT have been found to be non-toxic in mice and can be gradually excreted by the biliary pathway [28]. We explored the possibility of using supramolecular *π*–*π* stacking to load a cancer chemotherapy agent DOX on CNT/PPGP adducts for drug delivery applications.

In this work, we describe a previously unreported non-covalent CNT/PPGP/DOX supramolecular nano complex that can be developed for cancer therapy. We have investigated the ability of DOX to interact non-covalently and covalently with CNT functionalized with PPGP.

**Figure 9:**
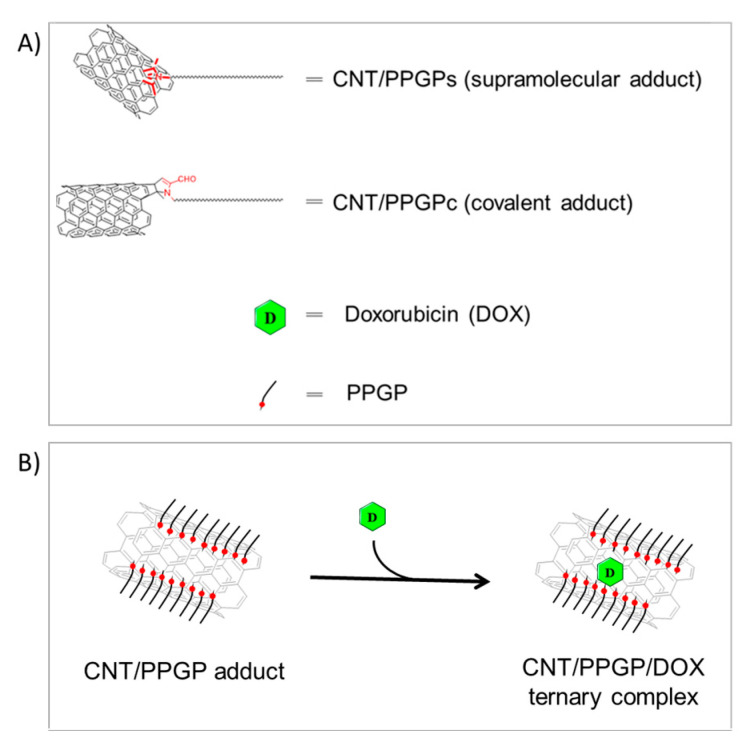
Representation of (**A**) the CNT/PPGP adducts, and (**B**) the hypothesized ternary complex CNT/PPGP/DOX.

Figure 9 shows schemes suggesting the structures of both covalent and supramolecular CNT/PPG adducts (Panel A) and the hypothesized ternary nano complex CNT/PPGP/DOX (Panel B).

The ternary nano complex was prepared, as described in Figure 9B. In brief, DOX hydrochloride was stirred for 16 h at room temperature with the modified nanotubes dispersed in a pH 7.4 PBS buffered solution. The CNT/PPGP/DOX nano complexes were isolated by repeated ultracentrifugation with PBS until the supernatant became colorless. Free, unbound DOX in the CNT supernatant was analyzed by UV-Vis spectroscopy. DOX characteristic absorbance peak at 490 nm was detected Figure S2. The amount of unbound DOX onto the CNT was estimated by measuring the absorbance at 490 nm relative to a calibration curve recorded under the same conditions Figure S2. In Table S1, the amount of loaded DOX is reported for pristine and functionalized CNT. After DOX loading on the PPGP modified carbon nanotubes, two different scenarios were observed for MWCNT and SWCNT respectively: (i) in MWCNT/PPGP/DOX UV-Vis spectrum the absorption band at 490 nm was not detected; (ii) in SWCNT/PPGP/DOX the absorption band at 490 nm is slightly redshifted (Figure S3).

**Figure 10:**
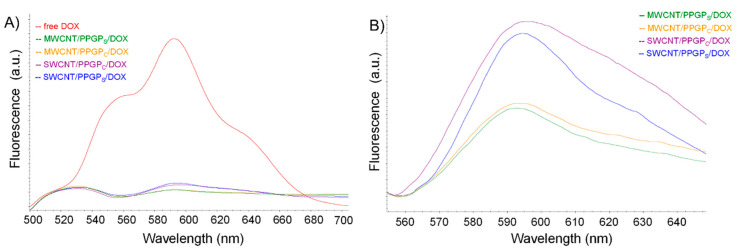
Normalized fluorescence intensities spectra of: (Panel **A**) DOX and CNT/PPGP/DOX nano complexes; (Panel **B**) CNT/PPGP/DOX nano complexes, zoom in the region between 560 to 640 nm. Irradiation at 480 nm.

The interaction between DOX and CNT/PPGP adducts was studied by monitoring the emission spectrum of DOX by fluorescence spectrophotometry (Figure 10).

As can be seen from Figure 10, the fluorescence quenching of DOX was evident for all the nano complexes.

**Figure 11:**
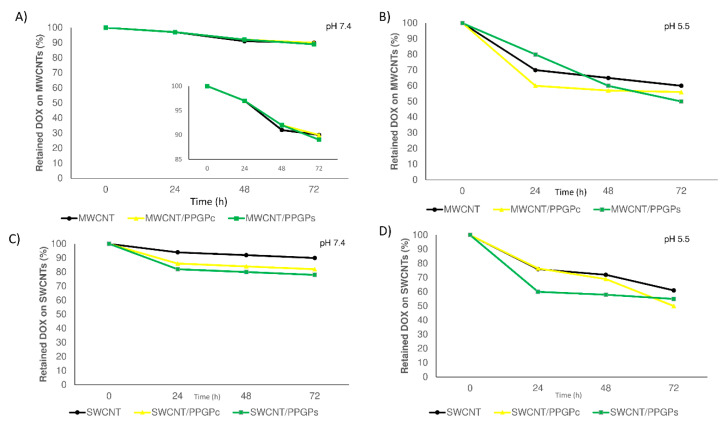
Drug release of the unmodified CNT/DOX and modified CNT/PPGP/DOX nano complexes in PBS buffer at 37 °C at pH 7.4 (**A**,**C**), MWCNT_s_ and SWCNT_s_ respectively) and in acetate buffer at pH 5.5 (**B**,**D**), MWCNTs and SWCNTs respectively); magnification in the range from 85% to 100% of retained DOX on MWCNT is also reported in the y-axis (**A**).

Release profiles of DOX (Figure 11) from all the modified and unmodified CNT at 37 °C were evaluated for up to 72 h at pH 7.4 in PBS (Figure 11A,C), which mimics the acidity of cytoplasm, and at pH 5.5 acetate buffer (Figure 11B,D), which mimics the acid condition of lysosomes, endosomes and cancerous tissues [47]. A slow-release with a pH-sensitive profile for all the investigated samples was observed. Our results were found to be in line with previous studies [48]. At physiological pH, DOX tended to remain bound to the CNT or CNT/PPGP adducts, whereas at acidic pH, the increased protonation of DOX changes both its solubility and hydrophilicity, hence leading to a higher release of the drug from the complexes [49].

No initial burst effect was observed in either of the conditions adopted. After 72 h, at pH 7.4 the amount of released DOX was in the range of 10–11% for MWCNT/DOX and MWCNT/PPGP/DOX complexes Figure 11A and of 10–20% for SWCNT/DOX and SWCNT/PPGP/DOX (Figure 11C). At pH 5.5, a different behavior was observed for CNT/DOX and CNT/PPPG/DOX complexes. Regarding MWCNT/DOX and MWCNT/PPGP/DOX complexes (Figure 11B), the amount of DOX released over a 72-h period was observed to be faster for MWCNT/DOX and MWCNT/PPGP_c_/DOX, while for MWCNT/PPGP_s_/DOX a controlled and a linear release was observed. In contrast, for SWCNT/DOX and SWCNT/PPGP/DOX complexes, the release of DOX at pH 5.5 Figure 11D was found to be faster for MWCNT/PPGP_s_/DOX and lower for MWCNT/DOX and MWCNT/PPGP_c_/DOX. The different complexes CNT/PPGP/DOX could satisfy different release rates, depending on the type of CNT and the nature of the interaction with PPGP and DOX.

### 3.3. Cell Viability Assay

In order to estimate the likelihood of our CNT drug delivery systems, the cytotoxicity of MWCNT, MWCNT/PPGP_s_, MWCNT/PPGP_c_, MWCNT/DOX, MWCNT/PPGP_s_/DOX, and MWCNT/PPGP_c_/DOX on A549 and M14 cell lines was evaluated. A stock dispersion of each CNT was prepared in culture medium at 1 mg/mL and sonicated. This procedure was ineffective for SWCNT, SWCNT/PPGP_s_, SWCNT/PPGP_c_, SWCNT/DOX, SWCNT/PPGP_s_/DOX, and SWCNT/PPGP_c_/DOX samples because of the persistence of carbon aggregates and agglomerates turned into the absence of a homogenous dispersion suitable for evaluation in cell culture assay. Indeed, the stock dispersion of MWCNT, MWCNT/PPGP_s_, MWCNT/PPGP_c_, MWCNT/DOX, MWCNT/PPGP_s_/DOX, and MWCNT/PPGP_c_/DOX was further diluted considering the amount of loaded DOX into each sample, as reported in Table S1. Thus, the concentrations were normalized and reported as the DOX amount.

**Figure 12:**
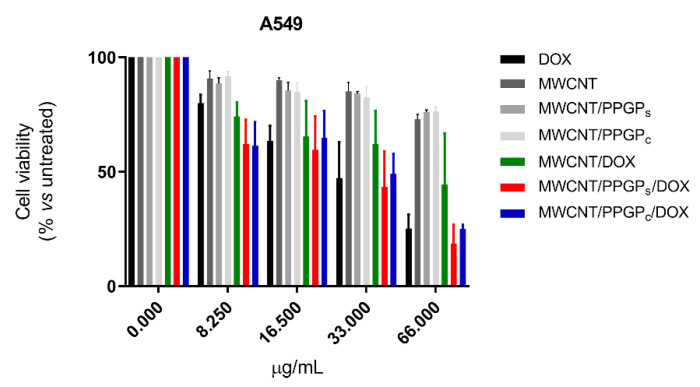
Cell viability (MTT assay) of A549 human melanoma cells untreated and treated for 48 h with different concentrations of free DOX hydrochloride or into both unloaded and loaded CNT. Each point represents mean ± s.e.m. of three separate experiments performed in triplicate.

**Figure 13:**
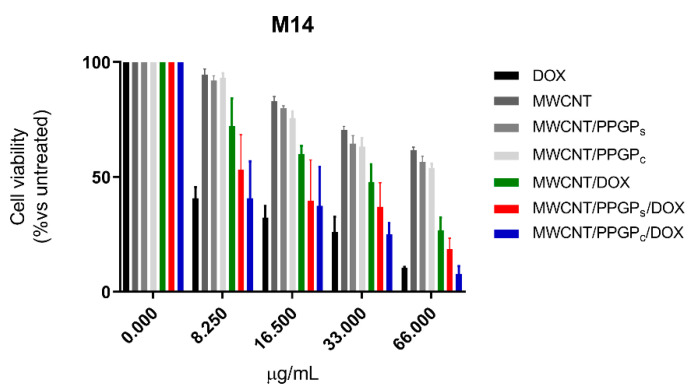
Cell viability (MTT assay) of M14 human lung adenocarcinoma cells untreated and treated for 48 h with different concentrations of free DOX hydrochloride or into both unloaded and loaded CNT. Each point represents mean ± s.e.m. of three separate experiments performed in triplicate.

The cytotoxic properties of loaded DOX were compared with that of the free DOX (Figure 12 and Figure 13) at four different concentrations (8.25 µg/mL, 16.5 µg/mL, 33 µg/mL, 66 µg/mL) after 48 h of treatment. Our preliminary results indicated that DOX maintains its inhibitory effect for all the investigated cases (free DOX, covalently loaded DOX, or complexed DOX) on both A549 and M14 cellular lines. Moreover, from our data, it emerges that there is a different behavior for the CNT drug delivery systems with a cell variance. MWCNT/DOX showed lower activity compared to the free DOX on M14 cellular lines; MWCNT/PPGP_c_/DOX and the free DOX displayed a similar effect on both cellular lines at all the investigated concentration; MWCNT/PPGP_c_/DOX appeared more efficient than free DOX in A549 cell lines and less efficient than the free DOX in MT14 ones.

Notably, the comparable cytotoxicity effects between free DOX and loaded DOX were discussed without considering the delayed release of DOX from the carrier. Considering the amount of DOX released at 48 h (about 35–43% at pH 5.5, Figure 11B, and 8–9% at pH 7.4, Figure 11A), a major cytotoxic effect can be ascribed to the DOX loaded on CNT respected to the free DOX, presumably due to a more efficient internalization route. Further investigation will be devoted to the studies of cellular uptake and intracellular trafficking of our nanocarrier to verify the chance to extend the use of this DDS.

### 3.4. Computational Studies

Although some molecular dynamics (MD) studies upon DOX/SWCNT systems were already performed [50,51,52,53,54,55], to the best of our knowledge, this is the first one that takes in consideration even a DOX/MWCNT system and, in particular, the use of opportunely reduced systems which reflects the actual diameter of the CNTs employed for the reported experiments and the CNT/PPGP/DOX ratios obtained from the experimentally observed weight percentages. So, we built four supramolecular systems named SSy1–4 corresponding to the complexes SWCNT/PPGP_s_/DOX, SWCNT/PPGP_c_/DOX, and MWCNT/PPGP_s_/DOX, MWCNT/PPGP_c_/DOX, respectively, and submitted each of them to 100 ns MD simulations. For each experiment, we used three different systems in which the starting positions of the PPGP and/or DOX molecules were randomly varied.

**Figure 14:**
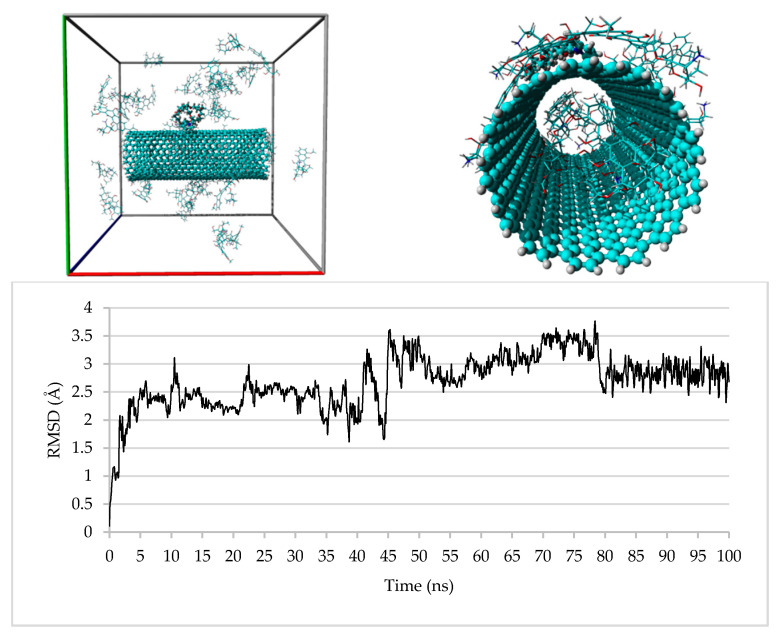
Upper: SSy2 at the start (left) and the end (right) of the MD simulation; bottom: plot of the RMSD of the DOX molecules during the 100 ns of the MD simulation.

**Figure 15:**
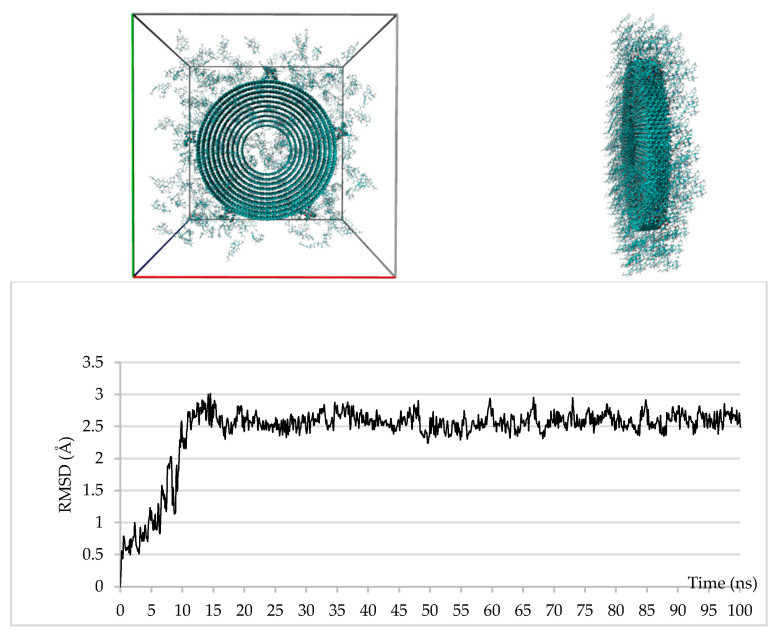
Upper: SSy4 at the start (left) and the end (right) of the MD simulation; bottom: plot of the RMSD of the DOX molecules during the 100 ns of the MD simulation.

The studied systems have reached their equilibrium states after about 10 and 15 ns of simulation time for the SSy1,2 and SSy3,4, respectively, as revealed by the root-mean-square displacements (RMSD) of DOX molecules reported, only for SSy2,4, in Figure 14 and Figure 15; it is evident that the fluctuations in RMSDs have reduced significantly after these periods.

**Figure 16:**
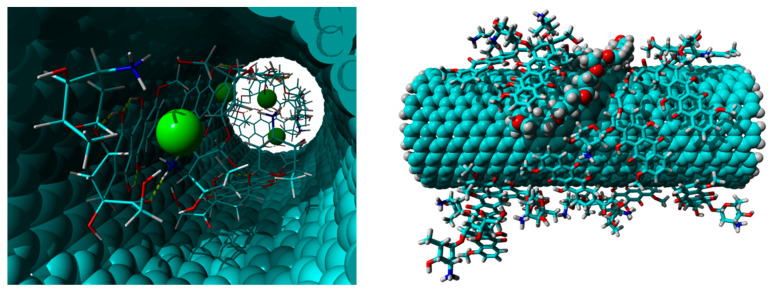
Left: view of the DOX molecules and the chloride ions contained inside the SWCNT cavity of the SSy2 at the end of the 100 ns of the MD simulation; right: view of the DOX molecules and the PPG pendant anchored to the external wall of the SWCNT of the SSy2 at the end of the 100 ns of the MD simulation.

In particular, Figure 14 shows the run of SSy2 in which there are two DOX molecules within the CNT at the starting time (0 ns) and 6 DOX molecules at the end time (100 ns). The entrance of the other four molecules, in pairs, takes place at 41.9 and 78.5 ns, respectively, as evidenced by the fluctuations registered in the RMSD graph for the DOX molecules. Interestingly, the six molecules within the CNT cavity were paired to four chloride ions, which coordinates some ammonium groups, some of which were facing each other (Figure 16), left; no sodium ion is present. The remaining DOX molecules are strongly anchored to the external wall of the SWCNT employing *π*–*π* interactions and, in some cases, two and even three of them are stacked together; the PPG pendant remains, most of the time, close to the external wall of the CNT (Figure 16, right).

As regards the SSy4 system, at the end of the 100 ns of the MD simulations, there are 8 DOX molecules within the inner CNT cavity, whereas almost all others are mainly adsorbed upon the external CNT surface of the far wall, stacked up to 5 units. Due to the short length of the tube (10 Å), the same DOX molecules are scattered along the two ends, usually with the most extended axis parallel to that of the CNT and stacked on each other.

The two different accommodation of DOX molecules between the two SSy2 and SSy4 systems are in accord to their respective side surface extensions, 1385 Å^2^ and 1492 Å^2^, except for that occupied by the covalently bound PPGP molecules. Considering that the semi-surface of a DOX molecule corresponds about to 75 Å^2^, about 18 upon 45, and 20 upon 354 DOX molecules for the SSy2 and the SSy4 system, respectively, should be able to cover the entire surface of each CNT. This also means that, on the equal surface, the efficiency of the MWCNT is approximately 7.8 times higher than that of the SWCNT. Finally, the ratio of about 1:5 of the PPGP molecules on the equal surface makes the MWCNT much more soluble than the SWCNT.

For the other two systems, SSy1,3, the trend is almost superimposable upon that of the corresponding systems with the covalently linked PPGP moieties. In both cases, the PPGP molecules are found to be sufficiently adherent to the surface of the CNT, mostly thanks to the portion of the PPG moiety; this is in accord with the TEM micrographs C,c and A,a of Figure 8 that highlight the presence of a polymeric layer adhered to the CNT surface. Moreover, for the SSy3 complex, the presence of 12 PPGP molecules strongly adsorbed on its surface make it even more soluble as the SSy4 parent.

## 4. Conclusions

Nanomedicine and technological nano delivery systems are a rather new but rapidly developing field where molecules in the nanoscale range are employed as diagnostic tools or to deliver therapeutics to specifically targeted sites and with a huge controlled fashion [56,57,58,59]. CNT are emerging nanomaterials with massive potential in the diagnostic and therapeutic fields. An effective way to make CNT more biocompatible and to increase their application in medicine is to functionalize the nanotubes. In this paper, we reported the functionalization of single and multi-walled carbon nanotubes with a pyrrole polypropylene glycol derived compound exploiting a *Diels*-*Alder* reaction. Thermogravimetric analysis and FT-IR spectroscopy showed that the functionalization of the considered adduct with PPGP was successful and that the introduction of a proper amount of modifier was achieved. WAXD shows that the functionalization procedures do not substantially alter per se the bulk structure of carbon nanotubes. The obtained functionalized CNT were then exploited to make a non-covalent CNT/PPGP/DOX supramolecular nano complex. The ability of DOX to interact with the non-covalent and the covalent PPGP modified CNT was investigated by experimental and computational techniques. HR-TEM microscopy confirmed that the covalent adducts show a regular adherent polymeric layer, and the supramolecular adducts contain on their surface both micelle-like structures and adherent polymer chains. MD simulations showed that DOX molecules can be adsorbed to the external wall of the nanotubes or included in their cavity.

Biological studies revealed that the in vitro activity of MWCNT/PPGP_s_/DOX and MWCNT/PPGP_c_/DOX are similar to that of the free DOX in A549 and M14 cell lines, although the former activities are actually attributable to a release, at 48 h, of approximately 8% (at pH 7.4) or 40% (at pH 5.5) of DOX.

Moreover, our studies show a different biological behavior between pyrrole functionalized-SWCNT and pyrrole functionalized-MWCNT, although a similar degree of chemical was detected for both materials. The formation of carbon aggregates and agglomerates in biological media for pyrrole functionalized-SWCNT prevented their evaluation, whereas the better dispersibility of pyrrole functionalized-MWCNT allowed the evaluation of their cytotoxicity in cell culture assay.

The use of carbon nanotubes in the drug delivery field seems promising due to the ability of CNT to cross biological barriers. This work paves the way for the facile functionalization of carbon nanotubes exploiting the “pyrrole methodology” for the development of novel technological carbon-based drug delivery systems.

Even if the preliminary biological studies were satisfactory, more mechanistic work is needed to investigate the capabilities of the novel “pyrrole functionalized” CNT to translocate into cells. 

Moreover, the intracellular trafficking of MWCNT/PPGP_s_/DOX, MWCNT/PPGP_c_/DOX, and released DOX that determines the drug efficacy and the related side effects also need be studied.

## Figures and Tables

**Table 1:** Mass losses for CNT and CNT/PPGP adducts from TGA analysis.

Samples	Mass Loss (%)		
	0 < T < 150 °C	150 < T < 700 °C	700 < T < 900 °C
SWCNT ^a^	1.6	1.0	97.4
SWCNT/PPGP_s_^a,b^	2.2	5.4	92.4
SWCNT/PPGP_c_^a,c^	0.1	5.0	94.9
MWCNT ^d^	2.0	0.2	97.8
MWCNT/PPGP_s_^b,d^	0.2	3.5	96.3
MWCNT/PPGP_c_^c,d^	0.1	8.0	91.9

^a^ Single-wall carbon nanotube; ^b^ supramolecular adduct; ^c^ covalent adduct; ^d^ multiwall carbon nanotubes.

**Table 2:** Degree ^a^ and yield ^b^ of functionalization of CNT functionalized with PPGP.

Adduct	Degree of Functionalization (%)	Functionalization Yield (%)
SWCNT/PPGP_s_	5.5	75
SWCNT/PPGP_c_	5.0	70
MWCNT/PPGP_s_	3.0	56
MWCNT/PPGP_c_	7.0	84

^a^ Calculated through Equation (1); ^b^ calculated through Equation (2).

## Supplementary Materials

The following are available online at https://www.mdpi.com/2079-4991/10/6/1073/s1, WAXD, HSP, and MD simulations details, Figure S1: ^1^H NMR spectrum (400 MHz, CDCl_3_) of the *O*-(2-(2,5-dimethyl-1*H*-pyrrol-1-yl) propyl)-*O*′-(2-methoxyethyl)polypropylene glycol (pyrrole polypropylene glycol, PPGP), Figure S2: UV-Vis absorbance spectra of unbonded DOX in PBS supernatant after centrifugation of: (A) MWCNT/DOX, MWCNT/PPGPc/DOX and MWCNT/PPGPs/DOX nano complexes; (B) SWCNT/DOX, SWCNT/PPGPc/DOX and SWCNT/PPGPs/DOX nano complexes. Figure S3: UV-Vis absorbance spectra of PBS solutions of free DOX (pink), MWCNT/PPGP/DOX covalent (yellow), and supramolecular (black) adducts, SWCNT/PPGP/DOX covalent (purple) and supramolecular (light blue) adducts. Figure S4: WAXD patterns of MWCNT (a), MWCNT/PPGP_s_ (b), and MWCNT/PPGP_c_ (c), Figure S5: TEM micrographs at low magnifications of pristine CNT and PPGP adducts, Table S1: Drug loading of the pristine and modified CNT, Table S2: Results of the inspections performed on the 1 mg/mL dispersions of the reported *sp*^2^ carbon allotropes (CA) in the listed solvents. The label ‘good’ indicates the stability of the dispersion, while the label ‘bad’ indicates a separation between the CA and the solvent. Section S1: Wide-Angle X-ray Diffraction Details. Section S2: Hansen Solubility Parameters (HSP) Details. Section S3: Molecular Dynamics Details.
