# Supplementary material for: Functionalization of Single and Multi-Walled Carbon Nanotubes with Polypropylene Glycol Decorated Pyrrole for the Development of Doxorubicin Nano-Conveyors for Cancer Drug Delivery
Source: Nanomaterials (Basel). 2020 May 31;10(6):1073. doi: 10.3390/nano10061073 (PMC7353207; doi:10.3390/nano10061073)
Supplement: Supplementary file 1 [file nanomaterials-10-01073-s001.pdf]

nanomaterials

Supplementary Information for

Functionalization of Single and Multi-Walled Carbon Nanotubes with Polypropylene Glycol Decorated Pyrrole for the Development of Doxorubicin Nano-Conveyors for Cancer Drug Delivery

Chiara Pennetta [1], Giuseppe Floresta [2], Adriana Carol Eleonora Graziano [3], Venera Cardile [3], Lucia Rubino[1], Maurizio Galimberti [1], Antonio Rescifina [2,*] and Vincenzina Barbera [1,*]

[1] Department of Chemistry, Materials and Chemical Engineering "G. Natta", Politecnico di Milano, Via Mancinelli 7, 20131 Milano, Italy; chiara.pennetta@polimi.it (C.P.); luciarita.rubino@polimi.it (L.R.); maurizio.galimberti@polimi.it (M.G.)

[2] Department of Drug Sciences, University of Catania, Viale Andrea Doria 6, 95125 Catania, Italy; giuseppe.floresta@unict.it (G.F.)

[3] Department of Biomedical and Biotechnological Science, Section of Physiology, University of Catania, Via Santa Sofia 97, 95123 Catania, Italy; acegraz@unict.it (A.C.E.G.); cardile@unict.it (V.C.)

* Correspondence: arescifina@unict.it (A.R.); vincenzina.barbera@polimi.it (V.B.); Tel.: +39-095-738-5014 (A.R.); Tel.: +39-02-738-4728 (V.B.)

S1. Wide-angle X-ray diffraction details

Distance between crystallographic planes was calculated from Bragg's law. The $D_{hk\ell}$ correlation length, in the direction perpendicular to the $hk\ell$ crystal graphitic planes, was determined to apply the *Scherrer* equation (Equation S1) where K is the *Scherrer* constant, λ is the wavelength of the irradiating beam (1.5419 A, Cu-K_α), $\beta_{hk\ell}$ is the width at half-height, and $\theta_{hk\ell}$ is the diffraction angle. The instrumental broadening, b, was determined by obtaining a WAXD pattern of a standard silicon powder 325 mesh (99%), under the same experimental conditions. The width at half height, $\beta_{hk\ell} = (B_{hk\ell} - b)$ was corrected, for each observed reflection with $\beta_{hk\ell} < 1°$, by subtracting the instrumental broadening of the closest silicon reflection from the experimental width at half height, $B_{hk\ell}$.

$$D_{hk\ell} = K\lambda/(\beta_{hk\ell} \cos\theta_{hk\ell}) \quad \text{Eq (S1)}$$

K is the Scherrer constant, λ is the wavelength of the irradiating beam (1.5419 Å, Cu-K_α), $\beta_{hk\ell}$ is the width at half-height, and $\theta_{hk\ell}$ is the diffraction angle. The instrumental broadening, b, was determined by obtaining a WAXD pattern of a standard silicon powder 325 mesh (99%), under the same experimental conditions. The width at half height, $\beta_{hk\ell} = (B_{hk\ell} - b)$ was corrected, for each observed reflection with $\beta_{hk\ell} < 1°$, by subtracting the instrumental broadening of the closest silicon reflection from the experimental width at half height, $B_{hk\ell}$.

S2. Hansen solubility parameters (HSP) details

The calculation of the cohesive energy density (U_T/V) of a compound as the sum of three interaction contributions: non-polar Van der Waals forces (δ_D), polar (δ_P), and hydrogen bonding (δ_H) as given by Equation S2.

$$\frac{U_T}{V} = \delta_T^2 = \delta_D^2 + \delta_P^2 + \delta_H^2 \quad \text{Eq (S2)}$$

The compound is therefore identified by three coordinates (δ_D, δ_P, and δ_H) in the Hansen Parameters space. The distance between two points (*e.g.*, a solute and its solvent) is related to their cohesive energy density difference, which is related to the enthalpy of mixing. As the enthalpy of mixing is minimal for miscible substances, two points close to each other in the Hansen space correspond to miscible compounds.

To estimate the HSP of a solute i, a dispersion test is performed on different solvents j, distinguishing suitable solvents (providing stable solutions/dispersions) and wrong solvents, which are not able to give stable dispersions. Given the parameters (coordinates) of the solvents, it is possible to define a sphere, centered on the solubility parameters of the solute, which encompasses the suitable solvents and excludes the non-solvents. The sphere radius is defined as R_0, the radius of interaction, while the distance between the solute and the solvent is R_a, calculated as in Equation S3.

$${R_{a,ij}}^2 = 4(\delta_{D,i} - \delta_{D,j})^2 + (\delta_{P,i} - \delta_{P,j})^2 + (\delta_{H,i} - \delta_{H,j})^2 \quad \text{Eq (S3)}$$

The ratio between $R_{a,ij}$ and R_0 is defined in Equation S4 as relative energy difference (RED). Solutes and solvents with good affinity have relative energy difference lower than 1 [1].

$$RED = \frac{R_{a,ij}}{R_o} \quad \text{Eq (S4)}$$

An optimization problem is therefore defined: the center coordinates of the Hansen solubility sphere are calculated by minimizing the radius of interaction (*i.e.*, the distance from the coordinates of the suitable solvents), including the suitable solvents (RED <1) and excluding the wrong ones. The sphere center coordinates correspond to the three unknown HSP of the solute.

The fitting sphere program was adapted from reference [2] and solved in the Matlab environment using the *Nelder-Mead* simplex algorithm.

S3. Molecular dynamics details

After prepared the systems as reported in paragraph 2.8.2. in the main text, the box was filled with the appropriate number of DOX molecules, randomly oriented, and, finally, the box was filled with water, with a maximum sum of all water bumps of 1.0 Å, and a density of 0.997 g mL^{-1} with explicit solvent. YASARA's pK_a utility was used to assign pK_a values at pH 7.2 [3], and the cell was neutralized with NaCl (0.9% by mass); in these conditions, 14 and 116 chlorine anions were used to neutralize the protonated amine groups (pK_a = 8.6, [4]) of the DOX molecules). Water molecules were deleted to readjust the solvent density to 0.997 g/mL. The final system dimensions were approximately 70 × 70 × 70 $Å^3$ and 120.0 × 120.0 × 120.0 $Å^3$ for the SWCNT and MWCNT systems, respectively. The PPGP and DOX force field parameters were generated with the AutoSMILES utility [5], which employs semiempirical AM1 geometry optimization and assignment of charges, followed by the assignment of the AM1BCC atom and bond types with refinement using the RESP charges, and finally the assignments of second-generation general AMBER force field (GAFF2) atom types. The nanotube, DOX, and PPGA molecules were treated with the GAFF2. Optimization of the hydrogen bond network of the various PPGP/DOX complexes was obtained using the method established by Hooft et al. [6], to address ambiguities arising from multiple side-chain conformations and protonation states that are not well resolved in the electron density. A short MD was run only on the solvent. The entire system was then energy minimized using first a steepest descent minimization to remove conformational stress, followed by a simulated annealing minimization until convergence (<0.01 kcal/mol Å). The MD simulation was then initiated, using the NPT ensemble at 298 K and integration time steps for intramolecular and intermolecular forces every 1.25 fs and 2.5 fs, respectively. Finally, 100 ns MD simulations without any restrictions were conducted, and the conformations of each system were recorded every 250 ps. On the averaged structure of the last 3 ns frames, a second cycle of energy minimization, identical to the first, was applied.

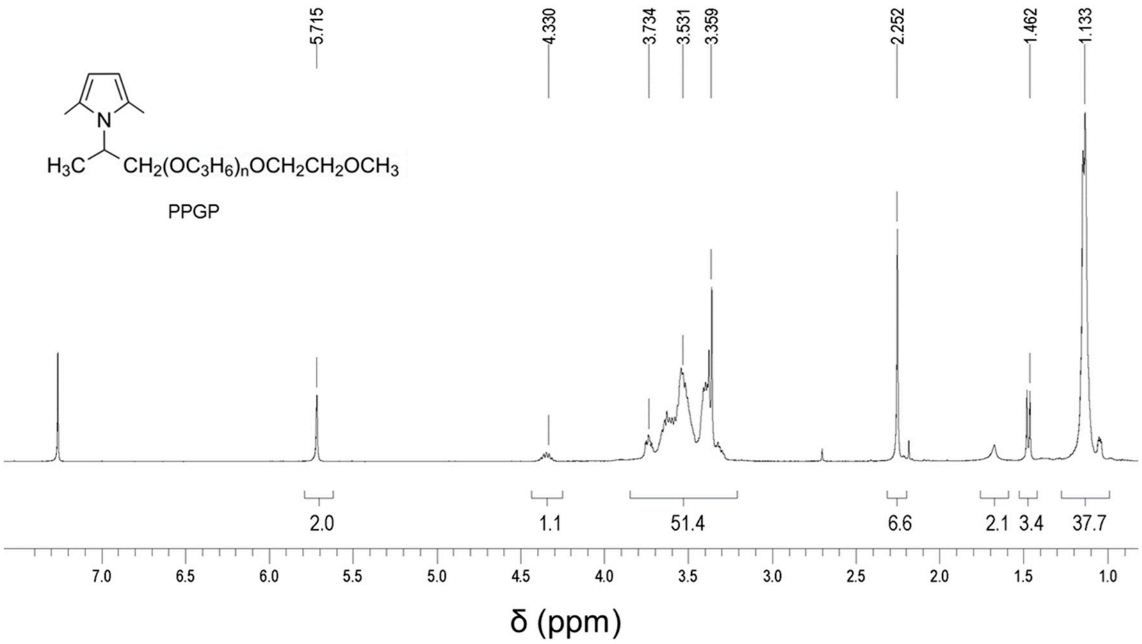

Figure S1. ^{1}H NMR spectrum (400 MHz, $CDCl_3$) of the *O*-(2-(2,5-dimethyl-1*H*-pyrrol-1-yl) propyl)-*O*′-(2-methoxyethyl)polypropylene glycol (pyrrole polypropylene glycol, PPGP).

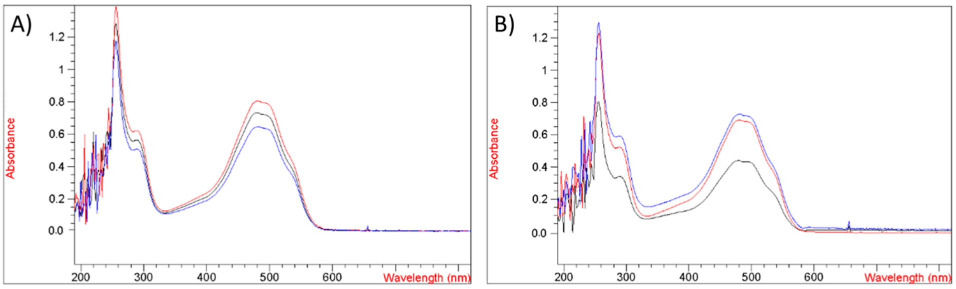

Figure S2. UV–Vis absorbance spectra of unbonded DOX in PBS supernatant after centrifugation of: (A) MWCNT/DOX, MWCNT/$PPGP_c$/DOX and MWCNT/$PPGP_s$/DOX nano complexes; (B) SWCNT/DOX, SWCNT/$PPGP_c$/DOX and SWCNT/$PPGP_s$/DOX nano complexes.

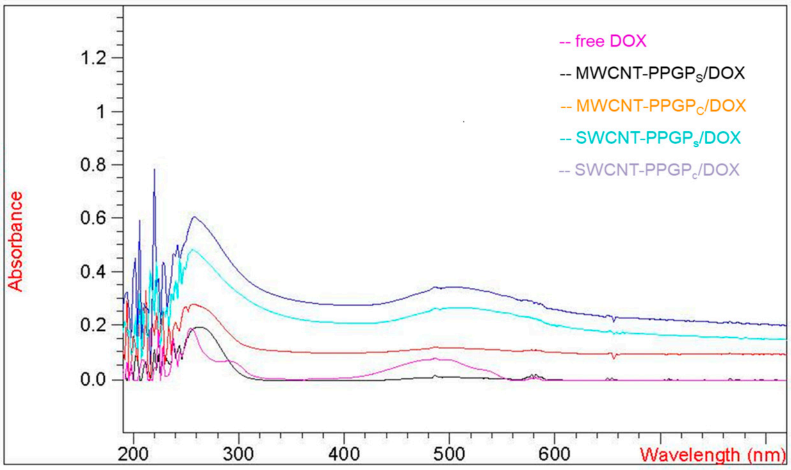

Figure S3. UV–Vis absorbance spectra of PBS solutions of free DOX (pink), MWCNT/PPGP/DOX covalent (yellow), and supramolecular (black) adducts, SWCNT/PPGP/DOX covalent (purple) and supramolecular (light blue) adducts.

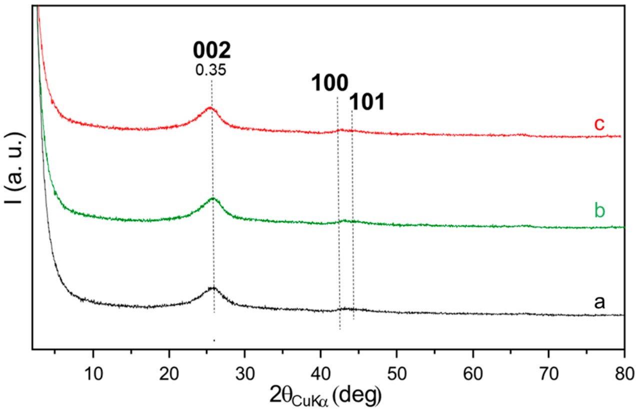

Figure S4. WAXD patterns of MWCNT (a), MWCNT/PPGP$_s$ (b), and MWCNT/PPGP$_c$ (c).

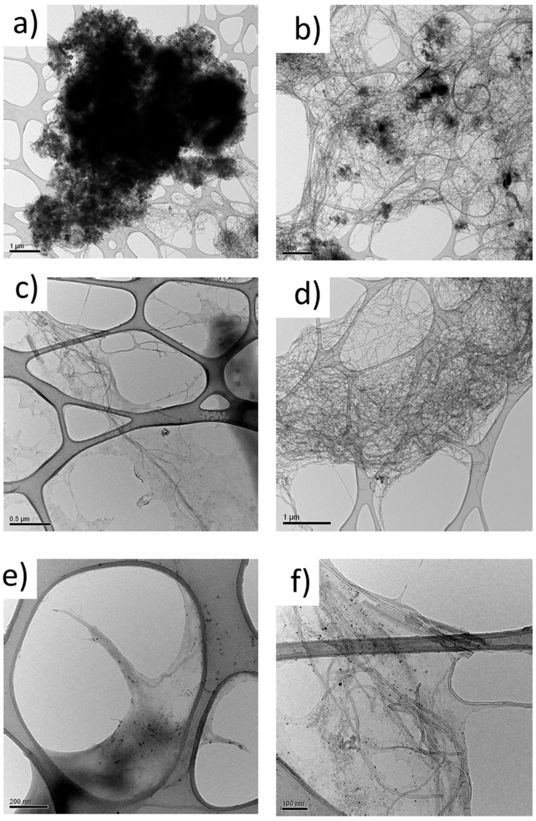

Figure S5. TEM micrographs at low magnifications of MWCNT (a), SWCNT (b), MWCNT/$PPGP_s$ (c), MWCNT/$PPGP_c$ (d), SWCNT/$PPGP_s$ (e) and SWCNT/$PPGP_c$ (f) adducts isolated from 1 mg/mL water dispersions.

Table S1. Drug loading of the pristine and modified CNT[a].

Carbon allotrope	DOX loading (w%)[b]
SWCNT	65.51
SWCNT/$PPGP_s$	66.39
SWCNT/$PPGP_c$	65.72
MWCNT	66.16
MWCNT/$PPGP_s$	65.62
MWCNT/$PPGP_c$	66.24

[a] The amount of unbound DOX onto the CNT was estimated by measuring the absorbance at 490 nm relative to a calibration curve recorded under the same conditions. Free, unbound DOX in the CNT supernatant was analyzed by UV-Vis spectroscopy. DOX characteristic absorbance band at 490 nm was detected (Figure S2) (see the experimental part in the main text); [b] weight percent of DOX hydrochloride loaded on 100 mg of CNT/PPGP/DOX nano complexes.

Table S2: Results of the inspections performed on the 1 mg/mL dispersions of the reported sp^2 carbon allotropes (CA) in the listed solvents. The label 'good' indicates the stability of the dispersion, while the label 'bad' indicates a separation between the CA and the solvent.

Solvent	MWCNT	MWCNT/$PPGP_c$
water	bad	good
methanol	bad	good
2-propanol	bad	good
acetone	bad	good
ethyl acetate	bad	good
propylene glycol	bad	good
dichloromethane	bad	good
xylene	bad	bad
toluene	bad	bad
hexane	bad	bad

References

1. Barbera, V.; Bernardi, A.; Palazzolo, A.; Rosengart, A.; Brambilla, L.; Galimberti, M., Facile and sustainable functionalization of graphene layers with pyrrole compounds. *Pure Appl. Chem.* **2018**, 90, 253-270.
2. Hansen, C. M., *Hansen solubility parameters: a user's handbook*. 2nd ed.; CRC Press: Boca Raton, 2007; p 519 p.
3. Krieger, E.; Nielsen, J. E.; Spronk, C. A.; Vriend, G., Fast empirical pKa prediction by Ewald summation. *J Mol Graph Model* **2006**, 25, 481-486.
4. Fritze, A.; Hens, F.; Kimpfler, A.; Schubert, R.; Peschka-Suss, R., Remote loading of doxorubicin into liposomes driven by a transmembrane phosphate gradient. *Biochimica Et Biophysica Acta-Biomembranes* **2006**, 1758, 1633-1640.
5. Jakalian, A.; Jack, D. B.; Bayly, C. I., Fast, efficient generation of high-quality atomic charges. AM1-BCC model: II. Parameterization and validation. *J Comput Chem* **2002**, 23, 1623-1641.
6. Krieger, E.; Dunbrack, R. L., Jr.; Hooft, R. W.; Krieger, B., Assignment of protonation states in proteins and ligands: combining pKa prediction with hydrogen bonding network optimization. *Methods Mol Biol* **2012**, 819, 405-421.
